# Sinoatrial Node Structure, Mechanics, Electrophysiology and the Chronotropic Response to Stretch in Rabbit and Mouse

**DOI:** 10.3389/fphys.2020.00809

**Published:** 2020-07-22

**Authors:** Eilidh A. MacDonald, Josef Madl, Joachim Greiner, Ahmed F. Ramadan, Sarah M. Wells, Angelo G. Torrente, Peter Kohl, Eva A. Rog-Zielinska, T. Alexander Quinn

**Affiliations:** ^1^Department of Physiology and Biophysics, Dalhousie University, Halifax, NS, Canada; ^2^Institute for Experimental Cardiovascular Medicine, University Heart Center Freiburg - Bad Krozingen, and Faculty of Medicine, University of Freiburg, Freiburg, Germany; ^3^Department of Electrical and Computer Engineering, Dalhousie University, Halifax, NS, Canada; ^4^School of Biomedical Engineering, Dalhousie University, Halifax, NS, Canada; ^5^Department of Physiology, Institut de Génomique Fonctionnelle, Montpellier, France

**Keywords:** mechano-electric coupling, heart rate, tissue stiffness, collagen, caveolae, second-harmonic generation microscopy, electron tomography, computational modeling

## Abstract

The rhythmic electrical activity of the heart’s natural pacemaker, the sinoatrial node (SAN), determines cardiac beating rate (BR). SAN electrical activity is tightly controlled by multiple factors, including tissue stretch, which may contribute to adaptation of BR to changes in venous return. In most animals, including human, there is a robust increase in BR when the SAN is stretched. However, the chronotropic response to sustained stretch differs in mouse SAN, where it causes variable responses, including decreased BR. The reasons for this species difference are unclear. They are thought to relate to dissimilarities in SAN electrophysiology (particularly action potential morphology) between mouse and other species and to how these interact with subcellular stretch-activated mechanisms. Furthermore, species-related differences in structural and mechanical properties of the SAN may influence the chronotropic response to SAN stretch. Here we assess (i) how the BR response to sustained stretch of rabbit and mouse isolated SAN relates to tissue stiffness, (ii) whether structural differences could account for observed differences in BR responsiveness to stretch, and (iii) whether pharmacological modification of mouse SAN electrophysiology alters stretch-induced chronotropy. We found disparities in the relationship between SAN stiffness and the *magnitude* of the chronotropic response to stretch between rabbit and mouse along with differences in SAN collagen structure, alignment, and changes with stretch. We further observed that pharmacological modification to prolong mouse SAN action potential plateau duration rectified the *direction* of BR changes during sustained stretch, resulting in a positive chronotropic response akin to that of other species. Overall, our results suggest that structural, mechanical, and background electrophysiological properties of the SAN influence the chronotropic response to stretch. Improved insight into the biophysical determinants of stretch effects on SAN pacemaking is essential for a comprehensive understanding of SAN regulation with important implications for studies of SAN physiology and its dysfunction, such as in the aging and fibrotic heart.

## Introduction

The heart’s intrinsic pacemaker, the sinoatrial node (SAN), generates spontaneous action potentials (AP) through a system of coupled oscillators whose common output initiates each normal heartbeat. The rate of SAN firing, and, thus, cardiac beating rate (BR), is determined by multiple subcellular mechanisms whose interaction and mutual entrainment form a robust system that drives SAN automaticity (Jalife, 1984; Noble et al., 1992; Lakatta et al., 2010). SAN automaticity is modulated by multiple factors, including the autonomic nervous system and various endocrine, paracrine, and autocrine agents, allowing adaptation of BR to changes in physiological demand (MacDonald et al., 2017, 2020). The SAN also responds to altered hemodynamic load through the Bainbridge response: an increase in BR upon right atrial distention, which may help in matching cardiac output to venous return (Bainbridge, 1915). This response was initially thought to be neurally mediated; however, mechanically induced changes in BR have since been demonstrated in isolated heart, atria, SAN, and single SAN cells, indicating the involvement of mechano-sensitive mechanisms (Quinn and Kohl, 2020) intrinsic to pacemaker cells (Quinn and Kohl, 2012). Here, we investigate how structural and mechanical properties of the SAN relate to amplitude and directionality of stretch-induced changes in BR, including a comparison between rabbit and mouse as important species-specific differences may exist (Cooper and Kohl, 2005).

Pacemaker cells in the SAN are interspersed with fibroblasts and embedded within a matrix of fibrous connective tissue, predominately consisting of collagen and elastin (Monfredi et al., 2010; Ho and Sánchez-Quintana, 2016). SAN cells are smaller than surrounding atrial cardiomyocytes (Boyett et al., 2000), and their sarcolemma shows less pronounced invaginations at Z-disks, which are thought to convey a “spare membrane pool” that, together with sarcolemmal caveolae, allows working cardiomyocytes to adapt to mechanically induced changes in volume:surface ratio that arise as a consequence of aspect ratio dynamics during the cycle of contraction and relaxation (Dulhunty and Franzini-Armstrong, 1975; Kohl et al., 2003). Interestingly, SAN cells appear to have more caveolae than working cardiomyocytes (reports range from a twofold to fivefold difference; Masson-Pévet et al., 1980; Kordylewski et al., 1993; Burton et al., 2017). In ventricular cardiomyocytes, stretch-induced sarcolemmal membrane-incorporation of caveolae has been observed during myocardial stretch, both in rabbit (Kohl et al., 2003) and mouse (Pfeiffer et al., 2014). Caveolae have been shown to play important roles in spatial compartmentalization of proteins, ion channels, and G-protein complexes that contribute to automaticity of SAN cells (Lang and Glukhov, 2018). Yet whether their presence in SAN cells is affected by the mechanical environment is as yet unknown.

SAN stretch has been most widely studied in the rabbit (Quinn and Kohl, 2016), and the structure and composition of rabbit SAN are well established. The volume of rabbit SAN tissue is comprised of ∼50% extracellular matrix and fibroblasts and ∼50% pacemaker cells (Bleeker et al., 1980; Opthof et al., 1987) with pacemaker cells having diverse cell volumes, morphologies, and ion current densities (Verheijck et al., 1998; Monfredi et al., 2018). The central SAN contains a dense arrangement of small, spindle-shaped pacemaker cells, interwoven and embedded in a dense collagen network, whereas in peripheral regions, cell arrangement is more ordered with a large proportion of pacemaker cells oriented parallel to the *Crista* terminalis (CT; Bleeker et al., 1980). Mouse SAN also contains a compact central region with densely packed pacemaker cells. However, unlike rabbit, these cells are generally well aligned and oriented perpendicular to the CT (Liu et al., 2007). There is proportionately less extracellular matrix, and there are fewer fibroblasts in mouse SAN, comprising ∼25% of volume (Hao et al., 2011; Glukhov et al., 2015). In the mouse SAN periphery, cells are loosely packed and – like in the rabbit – arranged parallel to the CT (Liu et al., 2007).

Investigating and comparing the importance of structural and mechanical SAN properties for the chronotropic response to stretch using rabbit and mouse is favorable as they both have thin endo- and epicardial layers, comprised primarily of connective tissue with most of the thickness between the outer layers composed of a mix of pacemaker cells and connective tissue (Opthof et al., 1987; Verheijck et al., 2001), whereas larger animals have thicker endo- and epicardial covers (Opthof, 1988; Kalyanasundaram et al., 2019). Yet, although there is a robust increase in BR when stretch of the SAN is sustained over the entire cardiac cycle in most species (including human, dog, cat, rabbit, guinea pig, and zebrafish; summarized in Quinn and Kohl, 2012), the response to sustained SAN stretch in mouse differs, causing a variable response that includes decreases in BR (Cooper and Kohl, 2005). This is an important consideration as mouse is a commonly utilized model for studies of SAN electrophysiology and its regulation.

The reason(s) for this difference in the chronotropic response of the SAN to sustained stretch between mouse and other animals has remained elusive. It has been suggested that it may not be due to a fundamental difference in subcellular stretch-activated mechanism(s), but rather to the fact that the same mechanism(s) result in different outcomes in mice compared to other species (Cooper and Kohl, 2005). Specifically, it is thought that current flowing through cation non-selective stretch-activated channels (*I*_*SAC,NS*_) may generate varying outcomes due to species-specific differences in SAN AP morphology (Cooper and Kohl, 2005). The reasoning for this is as follows. Activation of *I*_*SAC,NS*_ in cardiac cells causes their membrane potential to be “pulled toward” the *I*_*SAC,NS*_ reversal potential (*E*_*SAC,NS*_), which is between -20 and 0 mV in cardiomyocytes, including SAN pacemaker cells (Guharay and Sachs, 1984; Craelius et al., 1988; Cooper et al., 2000). For those phases of the SAN AP during which their dynamically changing membrane potential is moving *toward E*_*SAC,NS*_ (i.e., during spontaneous diastolic depolarization and during early AP repolarization; see green panels in Figure 3A), activation of *I*_*SAC,NS*_ would be expected to *accelerate* the intrinsic change in membrane polarization, thus causing more rapid diastolic depolarization or faster early repolarization, thereby increasing BR. Contrarily, during phases of the SAN AP during which the membrane potential is moving *away* from *E*_*SAC,NS*_ (i.e., late AP upstroke and late repolarization; see pink panels in Figure 3A), *I*_*SAC,NS*_ activation would oppose the intrinsic change, resulting in a slowed upstroke or delayed final repolarization, thus potentially contributing to a decrease in BR. Consequently, the chronotropic response to stretch that is sustained over the entire SAN cycle would be expected to arise as the net effect of both positive and negative chronotropic contributions of *I*_*SAC,NS*_ during different phases of the SAN AP. Species-specific differences in SAN AP morphology affect this balance of positive and negative chronotropic contributions. In rabbit, periods with a potentially positive chronotropic effect dominate the SAN AP–*I*_*SAC,NS*_ interrelation. In mouse, in contrast, diastolic depolarization and AP plateau are both proportionately shorter than in rabbit and other species (Verheijck et al., 2001), such that periods during which *I*_*SAC,NS*_ could act to increase or decrease BR are near-evenly balanced. We, therefore, tested whether prolonging the plateau of mouse SAN AP by exposure to 4-aminopyridine (4-AP, a blocker of rapidly activating potassium currents important for early repolarization in mouse SAN cells; Golovko et al., 2015) would result in a more consistent response of mouse SAN BR to stretch rather than the previously reported variable outcome (Cooper and Kohl, 2005).

We further hypothesize that the chronotropic response to stretch may be influenced by structural and mechanical properties of the SAN and that dissimilarities between rabbit and mouse exist. To test this, we assessed maximum changes in BR of rabbit and mouse isolated SAN upon sustained stretch and compared them to measured SAN stiffness. This was combined with second-harmonic generation microscopy (SHGM) for visualization of collagen in stretched and non-stretched SAN to investigate structural components, such as collagen fiber alignment, crimp wavelength, and fiber tortuosity, that may account for interspecies differences in passive tissue mechanics, and to explore their potential relation to stretch-induced changes in BR. Finally, using electron tomography (ET), we examined stretch effects on SAN membrane ultra-structure that could potentially affect mechano-transduction by membrane tension or alter the integrity of signaling hubs, such as contained in sub-sarcolemmal caveolae.

## Materials and Methods

All experimental procedures were approved by the Dalhousie University Committee for Laboratory Animals or the local Institutional Animal Care and Use Committee in Freiburg, Germany (Regierungspraesidium Freiburg, X-16/10R) and followed the guidelines of the Canadian Council on Animal Care or German animal welfare laws and guidelines (TierSchG and TierSchVersV), compatible with Directive 2010/63/EU of the European Parliament on the protection of animals used for scientific purposes. Details of experimental protocols have been reported following the Minimum Information about a Cardiac Electrophysiology Experiment (MICEE) reporting standard (Quinn et al., 2011).

### SAN Isolation

Female rabbits (New Zealand White, 2.1 ± 0.2 kg) were euthanized by ear vein injection of pentobarbital (140 mg⋅kg^–1^), followed by rapid excision of the heart, aortic cannulation, and Langendorff perfusion (20 mL⋅min^–1^) with 37°C, carbogen (95% O_2_, 5% CO_2_) saturated Krebs-Henseleit solution (containing, in mM: 120 NaCl, 4.7 KCl, 24 NaHCO_3_, 1.4 NaH_2_PO_4_, 1.0 MgCl_2_, 1.8 CaCl_2_, and 10 Glucose, with an osmolality of 300 ± 5 mOsm and a pH of 7.40 ± 0.05). The atria were removed and placed in a bath of Krebs-Henseleit solution. Adult female mice (C57BL/6J, 8–12 weeks) were sacrificed by cervical dislocation, followed by rapid excision of the heart, which was placed in a bath with Krebs-Henseleit solution for removal of the atria. For both species, the atria were separated from the ventricles by cutting along the atrio-ventricular valve plane and pinned down at the appendages in a Sylgard-lined dish (DC 170; Dow Corning, Midland, United States) with the surface that normally rests against the ventricle facing up, taking care to not stretch the tissue. The SAN was exposed by incisions from the cut right atrio-ventricular junction along the frontal surface of the superior (cranial) and inferior (caudal) *Venae cavae* (SVC, IVC, respectively). The rabbit SAN was further trimmed by dissection along the auricular side of the medial edge of the CT to remove the large (>200 mg) atrial appendage and the (much smaller) interatrial septum. When cutting along the CT, mouse SAN has previously been shown to demonstrate irregularities in BR (Verheijck et al., 2001), so for the mouse, the much smaller (∼3 mg) right atrial appendage and the tiny inter-atrial septum were left in place.

### SAN Stretch

**FIGURE 1:**
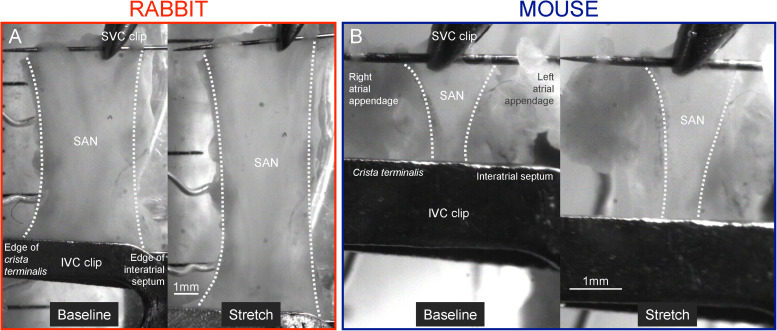
Isolated sinoatrial node (SAN) preparation. Representative images of isolated SAN before and during stretch from rabbit (40% stretch, **A**) and mouse (50% stretch, **B**). IVC, inferior (caudal) *vena cava*; SVC, superior (cranial) *Vena cava*.

Following SAN isolation, insect pins were woven through the cut edges of the SVC and IVC above and below the SAN tissue (Figure 1). A clip hanging from an isometric force transducer (PY2 72-4491, Harvard Apparatus, Holliston, United States) was attached to the SVC pin. A clip coupled to a computer-controlled linear DC-servomotor (LM 1247-02-01; FAULHABER MICROMO, Clearwater, United States) was attached to and supported the IVC pin with bipolar electrodes at either end of the pin connected to an ECG amplifier (Animal Bio Amp; ADInstruments, Colorado Springs, United States) for measurement of SAN electrical activity. This arrangement allowed for simultaneous stretch and measurement of force and BR (calculated from the peaks of the ECG or force signal; Supplementary Figure S1) in a water-jacketed imaging chamber, containing 37°C Krebs-Henseleit solution bubbled with carbogen.

After isolation and mounting, SAN preparations with irregular (*n* = 0) or low baseline BR (<290 beats⋅min^–1^) were excluded (*n* = 1 out of 14). The non-stretched SAN was lengthened in 10 μm steps of the linear motor until a baseline force of ∼0.2 g for rabbit (measured force = 0.21 ± 0.02 g) or ∼0.05 g for mouse (measured force = 0.07 ± 0.01 g) was applied to the tissue (based on preliminary experiments and previous studies by others (Kamiyama et al., 1984; Cooper and Kohl, 2005). Baseline tissue length (distance between the pins; 12.1 ± 0.6 mm for rabbit, 2.7 ± 0.2 mm for mouse) was measured as the distance between the SVC and IVC clips from video images (DMK 23UP1300; The Imaging Source, Charlotte, United States) with custom routines in Matlab (MathWorks, Natick, United States). Length was increased by 10% at a rate of 25 mm⋅s^–1^ with the linear motor, maintained for 30 s, and returned to baseline length at the same rate. After 120 s of rest, the 10% stretch/relengthening protocol was repeated once, followed by repetitions of the procedure with an increase in the amount of stretch by 10% for each iteration until either 50% stretch was applied or SAN BR became unstable (due to over-stretch).

Force and ECG signals were continuously recorded at 2 kHz using a data acquisition device (PowerLab, controlled by LabChart; ADInstruments). Force was measured in diastole (passive force) and converted to stress by dividing by the baseline cross-sectional area (perpendicular to the direction of stretch) of each SAN [rabbit = 1.45 ± 0.09 mm^2^, mouse = 0.28 ± 0.01 mm^2^; estimated as SAN width (measured from the video images with Matlab) multiplied by average SAN thickness for each species (294 μm for rabbit, 191 μm for mouse; measured from SHGM images of three isolated SAN from each species, embedded in agarose and sliced crosswise)]. Stretch was expressed as strain by dividing by baseline tissue length before stretch and values of BR and force from the two repeated stretches at each strain were averaged. An index of overall tissue stiffness (β) was calculated from the stress-strain data for each SAN by fitting the relationship: stress = α × e^β^ ^× strain^, where α indicates the *y*-axis intercept (Burkhoff et al., 2005; Garofalo et al., 2006).

### Experimental and Computational Modulation of Mouse SAN AP

SAN from mouse, prepared as described above, were subjected to two 30 s periods of 40% stretch, interspersed with 120 s of rest. 4-AP (275875; Sigma-Aldrich, St. Louis, United States; made freshly from a 25 mM stock solution in distilled water; pH titrated to 7.4 using HCl) was added to the chamber at final concentrations of 50, 100, and 500 μM, which had been shown previously to increase mouse SAN AP plateau duration without affecting the rate of diastolic depolarization or baseline BR (Golovko et al., 2015). After 2 min incubation at each of the target 4-AP concentrations as well as after a 20 min washout, the 40% stretch protocol was repeated.

The results of the above 4-AP experiments were further explored in computational simulations (Quinn and Kohl, 2013), using a mouse SAN cell AP model (Kharche et al., 2011). To simulate the electrophysiological effect of SAN stretch, a Hodgkin-Huxley formulation for an instantly activating, non-inactivating SAC_*NS*_ current with a linear current-voltage relationship as proposed by Healy and McCulloch (2005), was added to the model. This current was defined as *I*_*SAC,NS*_ = *g*_*SAC,NS*_ × (*V*_*m*_(t) - *E*_*SAC,NS*_), where *E*_*SAC,NS*_ = –10 mV (in keeping with SAN; Cooper et al., 2000). V_*m*_ is the time-varying membrane potential, and *g*_*SAC,NS*_ = 0.000248 nS/pF is the maximum whole cell SAC_*NS*_ conductance (which caused a 10% reduction in the absolute value of the maximum diastolic potential upon simulated cell stretch). Application of 4-AP was simulated by a reduction in the conductance of rapidly activating potassium currents in the model (with the transient outward potassium current set to zero and the delayed rectifier potassium current reduced by 25%). Simulations were run to steady state (10 s of simulated SAN activity) in Dev-C++, using a standard explicit Euler integration method with a constant time step of 0.1 ms.

### Collagen Imaging and Characterization in SAN Tissue

SAN from rabbit and mouse, prepared as above, were left at baseline length (*n* = 3 for each species) or subjected to 40% stretch (*n* = 3 for each species) and immediately fixed with 4% paraformaldehyde in phosphate-buffered saline. After 1 h, samples were washed in phosphate-buffered saline and used for imaging. Collagen was visualized by SHGM on an upright microscope (TCS SP8 DIVE; Leica Microsystems, Wetzlar, Germany) using a 25×, 1.0 NA, water immersion objective (IRAPO L 25x/1.00 W; Leica Microsystems) with 920 nm illumination from a pulsed laser (InSight X3 Dual; Spectra-Physics, Santa Clara, United States). 3-D imaging was performed by recording tiled z-stacks (442.9 × 442.9 μm^2^ in x-y, between 100 and 200 μm in z, with six to nine tiled z-stacks per SAN). Stitching and image processing were done in Leica LAS-X (Leica Microsystems).

Collagen orientation was analyzed in the central SAN at the epicardial surface and in the middle layers of SAN tissue by tracing the skeleton of 50 representative fibers in each of the acquired SHGM stacks, using the “Simple Neurite Tracer” (Longair et al., 2011) plugin within Fiji (Schindelin et al., 2012). To enable interactive tracing, image stacks were first laterally binned four times. Principal component analysis of each fiber was performed to define the principal eigenvectors with the largest eigenvalue, indicating fiber direction. Polar orientation plots displaying the measured fiber directions were then generated using the Python package Matplotlib (Hunter, 2007). For characterization of collagen fibers, crimp wavelength and fiber tortuosity were measured by a blinded observer using ImageJ (Schneider et al., 2012). Wavelength was measured as the linear distance between two adjacent collagen fiber wave peaks (Pierlot et al., 2014). Eight wavelength measurements were made in three SHGM images for each layer (surface or middle) for a total of 48 measurements per preparation (non-stretched or stretched in mouse and rabbit). Tortuosity was calculated as the ratio of the collagen path length traced directly along the fiber between two points divided by the linear distance between those points (Jan et al., 2018). Three tortuosity values were calculated in three images for each layer for a total of nine measurements per preparation.

### Caveolae Imaging and Characterization in SAN Cells

For ET imaging of sub-sarcolemmal caveolae, SAN isolated from rabbit or mouse (as described above) were cut longitudinally (from the SVC to IVC) into two strips. One strip was left non-stretched, and the other was stretched, resulting in a change in SAN pacemaker cell sarcomere length from 1.76 ± 0.03 to 2.16 ± 0.04 μm for rabbit and from 1.75 ± 0.02 to 2.09 ± 0.05 μm for mouse. Immediately after application of stretch, strips were immersion-fixed in iso-osmotic (300 mOsm) Karnovsky’s fixative (2.4% sodium cacodylate, 0.75% paraformaldehyde, 0.75% glutaraldehyde; Solmedia, Shrewsbury, United Kingdom). Tissue fragments were excised from fixed SAN strips and prepared for ET (Rog-Zielinska et al., 2016). Briefly, fragments were washed with 0.1 M sodium cacodylate, post-fixed for 1 h in 1% OsO_4_, dehydrated in graded acetone, and embedded in Epon-Araldite resin. Semithick (275 nm) sections were placed on formvar-coated slot-grids, poststained with 2% aqueous uranyl acetate and Reynold’s lead citrate. Colloidal gold particles (15 nm; BBI Solutions, Crumlin, United Kingdom) were added to both surfaces of the sections to serve as fiducial markers for tilt series alignment.

Imaging was performed in the Electron Microscopy Core Facility of the European Molecular Biology Laboratory (EMBL) in Heidelberg, using a transmission electron microscope (Tecnai TF-30 300 KV; formerly FEI Company, now Thermo Fisher Scientific, Waltham, United States) and a 4 K × 4 K charge-coupled device camera (OneView; Gatan, Munich, Germany). Isotropic voxel size was (1.55 nm)^3^. Double-tilt tomograms were processed and analyzed using IMOD software, which was also used to generate 3-D models of relevant structures. The number of sub-sarcolemmal caveolae per unit of cell surface area was calculated, and surface membrane convolution was assessed as the total surface area (including folds) relative to a flat projection.

### Statistical Analysis

Data are presented as mean ± standard error of the mean (SEM). Unpaired Student’s *t*-test and mixed-effects analysis or one-way ANOVA were used for comparison of means, when appropriate, and *post hoc* comparisons were performed using Sidak’s or Tukey’s multiple comparison test, respectively. Linear regression was used to assess the relationship between variables. A *p*-value of less than 0.05 was considered to indicate a statistically significant difference between means.

## Results

### Chronotropic Response to Stretch and Its Relation to SAN Stiffness

**FIGURE 2:**
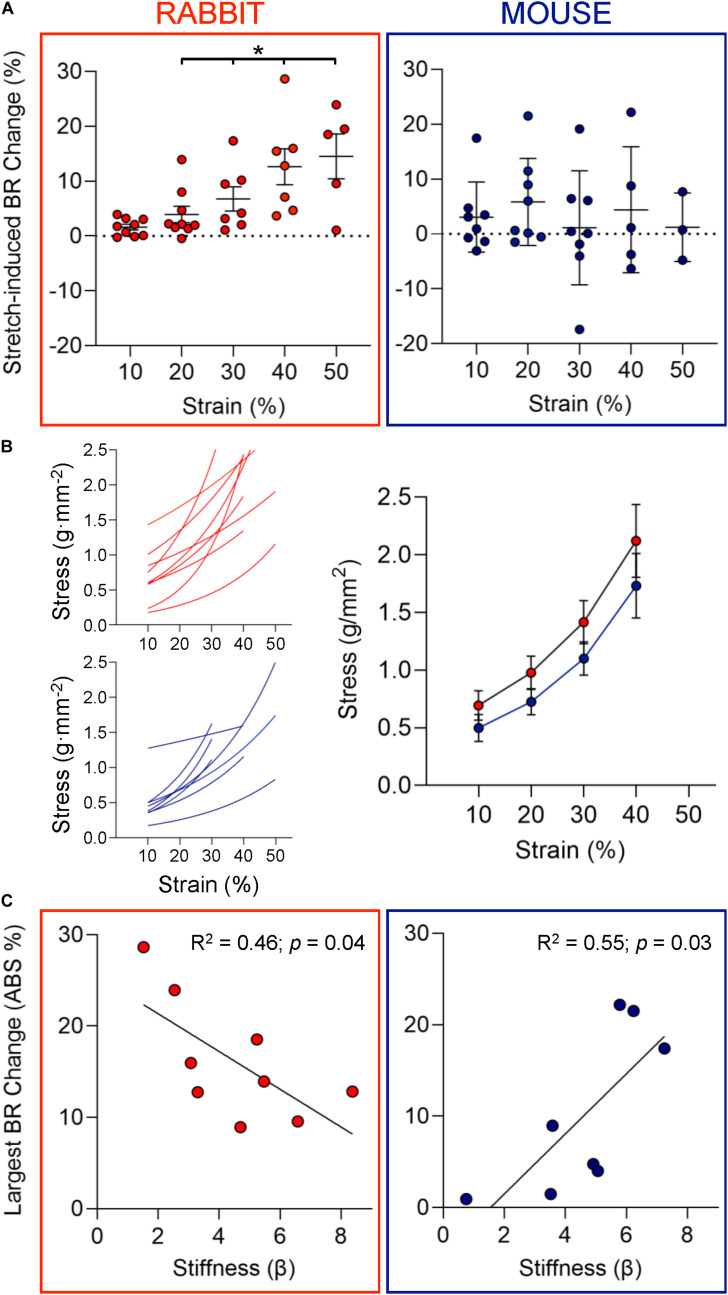
Chronotropic response to stretch and mechanical properties of SAN. **(A)** Change in beating rate (BR) of rabbit (left, *n* = 9) and mouse (right, *n* = 8) SAN upon strain application of increasing magnitude. **(B)** Individual and average stress-strain curves for rabbit (red) and mouse (blue) SAN. **(C)** The relationship between the largest absolute (ABS) percentage change in BR observed upon SAN stretch and tissue stiffness (β) of the individual sinoatrial node studied for rabbit (left) and mouse (right). *p < 0.05 indicates a significant increase in BR by mixed-effects analysis.

After isolation and mounting, all SAN demonstrated regular spontaneous beating. In the first set of experiments involving stretch in physiological saline only, baseline BR at the start of the experimental protocol in rabbit SAN was 192 ± 7 beats⋅min^–1^ (*n* = 9), and in mouse SAN, it was 407 ± 22 beats⋅min^–1^ (*n* = 8). Baseline BR did not change significantly throughout the entire experimental investigation (up to 2 h). Stretch of rabbit SAN caused an increase in BR at strain levels of 20% or greater (*p* < 0.05 by mixed-effects analysis) with BR returning to baseline between each stretch application and with the magnitude of the change in BR correlating with the amount of strain (Figure 2A and Supplementary Figure S2A). Stretch of mouse SAN resulted in a more variable chronotropic response: BR increased in three mice, decreased in one mouse, and varied between increase and decrease in four mice (Supplementary Figure S2B). There was no correlation between the magnitude of the stretch-induced change in BR and baseline BR prior to stretch application in either species (Supplementary Figure S3).

Individual stress-strain curves (whose slopes represent tissue stiffness) were generated for each SAN preparation. There was no difference between mean stress-strain behavior in rabbit and mouse (Figure 2B). To determine the relationship between the magnitude of electrophysiological responses to stretch and SAN stiffness, the largest *absolute* percentage change in BR observed in each individual SAN preparation was identified and plotted against SAN tissue stiffness (β, Figure 2C; note: absolute values of BR change were used to assess responsiveness independently of directionality of change). Interestingly, for rabbits, there was an inverse relationship between the chronotropic responsiveness to stretch and SAN stiffness although for mice the relationship was positive (*p* < 0.05 for both by linear regression).

### Effect of 4-AP on the Chronotropic Response to Stretch in Mouse

**FIGURE 3:**
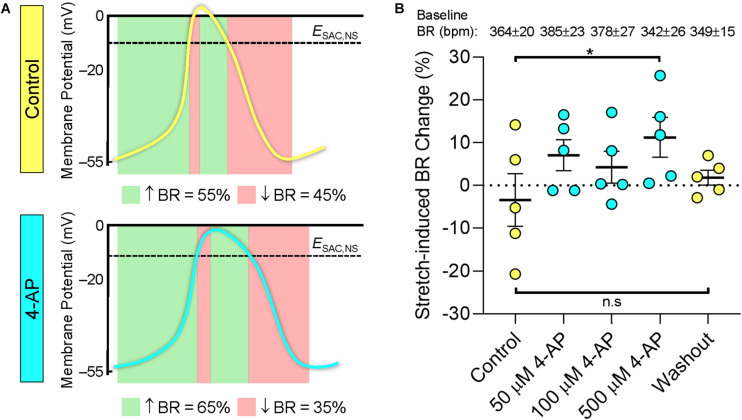
Relationship between action potential morphology and the chronotropic response to stretch of the mouse SAN. **(A)** Theoretical effects of stretch, sustained over the cardiac cycle, on BR of the mouse SAN in control conditions (top) and during application of 4-aminopyridine (4-AP, bottom). Green panels indicate phases during which activation of a cation non-selective stretch activated current (*I*_*SAC,NS*_) would accelerate intrinsic changes in SAN membrane potential and, hence, increase BR, and red panels indicate phases during which *I*_*SAC,NS*_ activation would decrease BR. Mouse action potentials redrawn from microelectrode recordings in Figure 2 from Golovko et al., 2015. **(B)** Experimentally observed changes in BR of mouse SAN upon 40% stretch before (Control), during (X μM 4-AP), and after (Washout) application of increasing levels of 4-AP (*n* = 5). Baseline BR in the absence of stretch for each concentration of 4-AP is indicated at the top of the figure (in beats⋅min^–1^, bpm). *E*_*SAC,NS*_, reversal potential of *I*_*SAC,NS*_. *p < 0.05 indicates a significant increase in BR compared to control by mixed-effects analysis; n.s. indicates no significant difference.

In further experiments (*n* = 5), 40% stretch of the mouse SAN again resulted in a variable chronotropic response with BR increasing in two of the SAN tested and decreasing in the other three (Figure 3B and Supplementary Figure S2C). After application of increasing concentrations of 4-AP, there was a shift in the stretch-induced change in BR toward a positive chronotropic response with a significant difference seen at a concentration of 500 μM (*p* < 0.05, mixed-effects analysis). After 20 min washout, there was no longer a difference in stretch-induced BR changes compared to the initial control. As in the first set of experiments, the magnitude of stretch-induced BR changes was not related to baseline BR before stretch (Supplementary Figure S4). Baseline BR was not affected by 4-AP (in agreement with previous findings in mouse SAN; Golovko et al., 2015) and did not change significantly over the course of the experiment.

In the computational simulations (AP presented in Supplementary Figure S5), stretch in control conditions resulted in a 10% decrease in BR (even though the rate of diastolic depolarization was increased by 20%), which is within the range of stretch-induced changes in BR seen in control experiments. Simulation of 4-AP application by reducing the magnitude of rapidly activating potassium currents resulted in a 32% increase in APD_20_ with little effect on BR (-1%) similar to previous reports (Golovko et al., 2015). In the presence of 4-AP, the negative chronotropic response to simulated stretch was nearly abolished (BR changed by -3% instead of -10% compared to prestretch values).

### SAN Microstructure and the Effect of Stretch: Collagen

**FIGURE 4:**
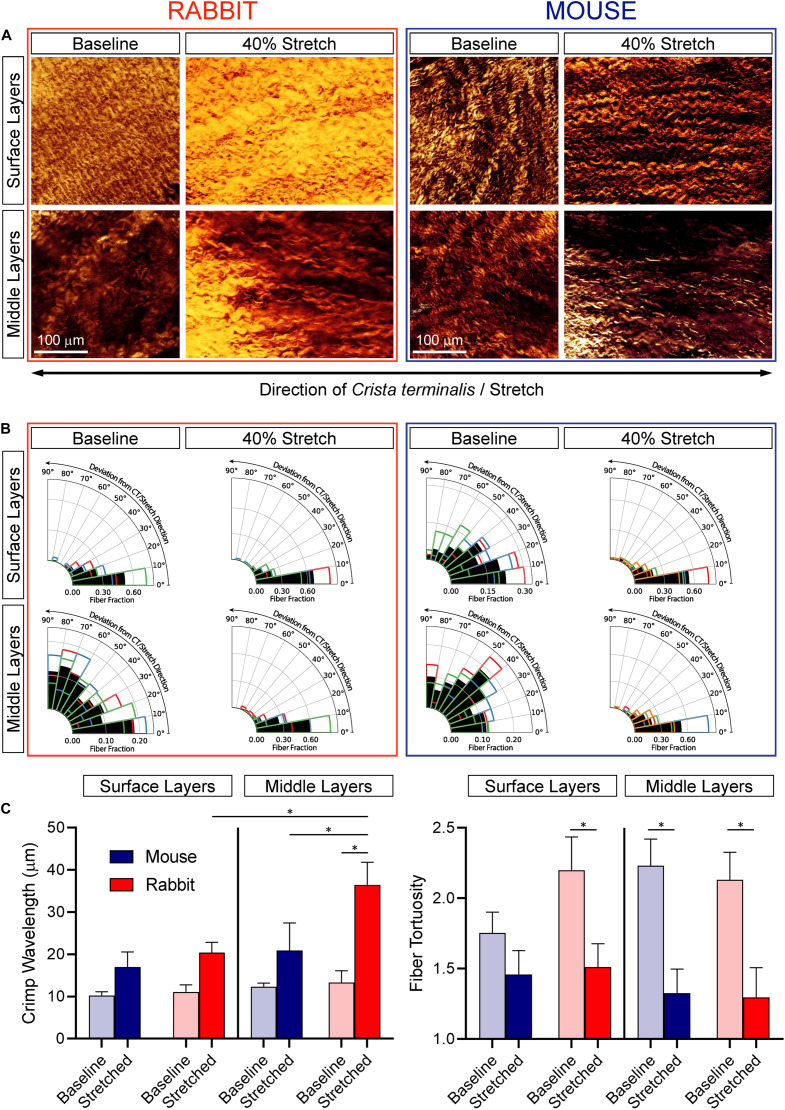
Effect of SAN stretch on collagen alignment and crimp characteristics. **(A)** Representative second-harmonic generation images of collagen at the epicardial surface and in the middle layers of the central SAN of rabbit (left) and mouse (right) at baseline and during 40% stretch. **(B)** Collagen orientation analysis for all samples (*n* = 3 for each). Black bars represent the fraction of fibers averaged across all samples and colored outlines represent the average fraction of fibers from individual samples. **(C)** Collagen crimp wavelength (left) and fiber tortuosity (right). *p < 0.05 indicates a difference between the means by one-way ANOVA.

Collagen was visualized with SHGM in both the surface and middle layers of the central SAN of rabbit and mouse at baseline and at 40% stretch (Figure 4A; *n* = 3, for both baseline and stretch in each species).

In the dense epicardial surface layers of non-stretched SAN, collagen fiber alignment differed between rabbit and mouse: fibers were highly aligned in parallel to the CT in rabbit but were less organized with a more distributed orientation in mouse (see surface layers at baseline in Figure 4B). During 40% stretch, collagen fibers were highly aligned parallel to the CT in the surface layers both of rabbit and mouse SAN. Thus, the less organized collagen fibers in the surface layers of mouse SAN rotated in the direction of stretch and became highly aligned with the CT. In both species, surface collagen was tightly crimped (as indicated by relatively low wavelength) and tortuous (Figure 4C). Counterintuitively, there was no difference in crimp wavelength between stretched and non-stretched SAN from either species, presumably indicative of a rotational reorientation of collagen that may precede stretching. Tortuosity was also not different in stretched versus non-stretched SAN from mouse but was lower in stretched versus non-stretched rabbit SAN (for which collagen rotation would be less pronounced than in mice as it is already aligned in parallel to CT before application of stretch).

In the less-dense middle layers of non-stretched SAN from both species, collagen fibers were disorganized (with a distributed orientation; Figure 4B), crimped, and tortuous (Figure 4C). In stretched SAN, collagen fibers rotated in the direction of stretch to become highly aligned with the CT (∼2–3-fold increase; Figure 4B), and they were less tortuous than in non-stretched SAN (Figure 4C). Crimp wavelength was not different between stretched and non-stretched SAN of the mouse but were greater in stretched compared to non-stretched rabbit SAN (Figure 4C). Crimp wavelength in stretched rabbit SAN was also greater in middle versus surface layers.

### SAN Nanostructure and the Effect of Stretch: Caveolae

**FIGURE 5:**
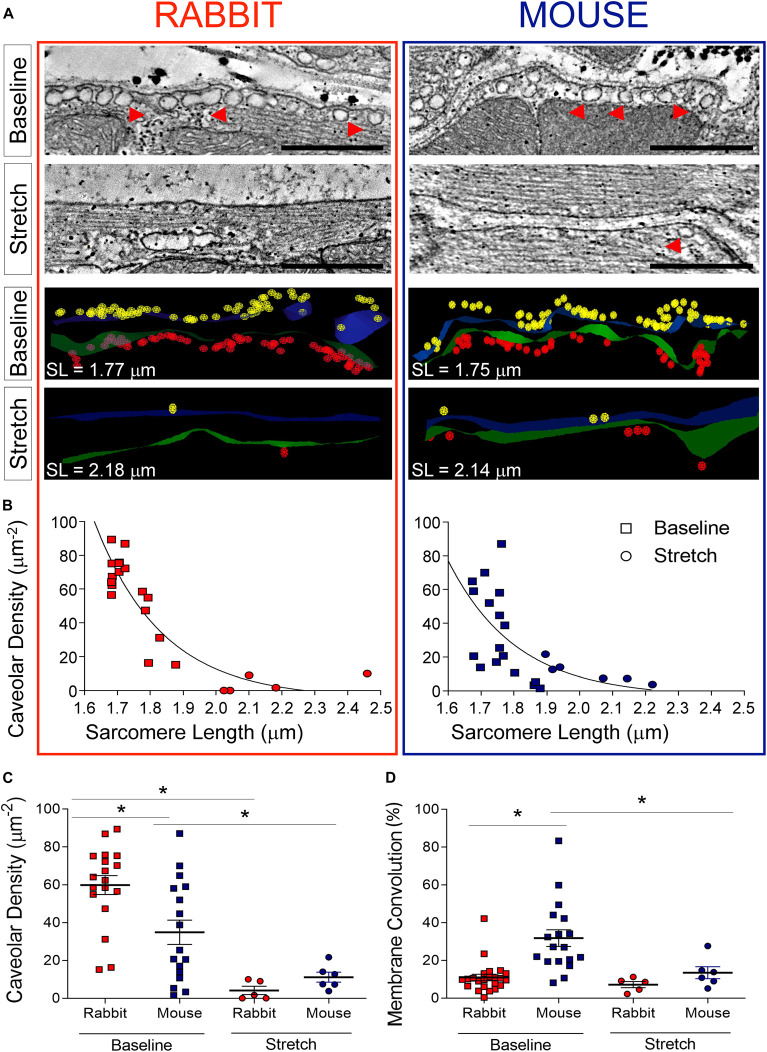
Effect of stretch on SAN nanostructure. **(A)** Representative electron tomography slices and three-dimensional segmentations of the sarcolemma and sub-sarcolemmal caveolae (examples indicated by red arrowheads) in rabbit (left) and mouse (right) SAN cells. **(B)** Relationship between caveolar density and sarcomere length (SL) across all preparations at baseline (square symbols; *n* = 18 cells for rabbit, *n* = 17 cells for mouse) and during stretch (round symbols; *n* = 5 cells for rabbit, *n* = 6 cells for mouse). **(C)** Average values of caveolar density for rabbit and mouse at baseline and during stretch. **(D)** Average values of surface membrane convolution for rabbit and mouse at baseline and during stretch. Green/blue, surface membranes of two opposing SAN cells; yellow/red, caveolae in the two cells. *p < 0.05 indicates a difference between the means, by one-way ANOVA.

Sub-sarcolemmal caveolae were abundant in SAN cells at baseline in both species (Figure 5A; *n* = 18 cells for rabbit, *n* = 17 cells for mouse), and there were nearly twice as many caveolae per unit of cell surface area in rabbit SAN than in mouse (*p* < 0.05 by one-way ANOVA; Figure 5C). Upon stretch, both in rabbit (*n* = 5 cells) and mouse (*n* = 6 cells) SAN, there was a significant reduction in the presence of caveolae with almost complete sarcolemmal membrane incorporation at sarcomere lengths ≥ 2 μm (Figure 5B; total membrane surface area analyzed was: baseline = 19.5 μm^2^, stretch = 3.2 μm^2^ for rabbit; baseline = 12.3 μm^2^, stretch = 6 μm^2^ for mouse). The difference in sub-sarcolemmal caveolae content between rabbit and mouse in SAN cells, observed at baseline, thus disappeared during stretch (Figure 5C).

Concurrently, the surface membrane was significantly more convoluted at baseline in mouse SAN cells compared to rabbit, providing an excess surface compared to a 2-D “flat projection” of the cell surface, of 10.9 ± 1.7% in rabbit versus 31.8 ± 4.4% in mouse (*p* < 0.05 by one-way ANOVA), a difference that was also abolished with stretch (Figure 5D).

Taking into account *both* membrane convolution and caveolae, the total “spare membrane” reserve at baseline (as compared to a 2-D flat projection) is higher in rabbit compared to mouse SAN cells (excess sarcolemmal surface at rest was 141 ± 3.7% in rabbit versus 118 ± 7.3% in mouse; *p* < 0.05, unpaired Student’s *t-*test).

## Discussion

In this study, we demonstrate (i) a correlation between SAN stiffness and the magnitude of the stretch-induced change in BR, which differs between rabbit and mouse; (ii) species differences in SAN tissue structure and sub-sarcolemmal caveolae density as well as their change with stretch; and (iii) a shift of stretch-induced changes in mouse SAN BR toward positive chronotropy in the presence of 4-AP.

### Species Difference in the Chronotropic Response to Stretch and Their Relation to SAN Stiffness

The stretch-induced increase in BR of rabbit SAN and the variable response of mouse SAN observed in the current study (Figure 2A) are consistent with previous reports (Deck, 1964; Golenhofen and Lippross, 1969; Ushiyama and Brooks, 1977; Kamiyama et al., 1984; Arai et al., 1996; Cooper and Kohl, 2005). Yet little is known about the mechanical determinants of the chronotropic response to SAN stretch. Although some previous studies have suggested that the amount of strain is a key input parameter (Sanders et al., 1979; Kamiyama et al., 1984), others have proposed that stress levels (Brooks et al., 1966; Chiba, 1977; Arai et al., 1996) or a combination of both (Lange et al., 1966), determine mechanical effects on BR.

We observed that the largest absolute changes in BR upon stretch show an inverse correlation with SAN tissue stiffness in rabbit (i.e., the least stiff SAN preparations show the largest responses) although in mouse there is a positive correlation (i.e., the stiffest SAN respond most; Figure 2C). This suggests that rabbit SAN pacemaker cells may respond primarily to strain, and mouse SAN pacemaker cells may be more sensitive to stress. This would mean that effects of mechanical loading on SAN BR may depend on the mode of load application (i.e., whether a specific magnitude of stretch or a specific magnitude of force is applied), perhaps explaining some of the previous lack of agreement between experimental studies. One caveat of all these studies (including ours) is that sustained mechanical stimulation has been used, applied throughout systole and diastole. This differs from the *in vivo* setting, in which the right atrium is cyclically stretched during atrial diastole by ventricular contraction (valve plane shift) and accumulation of venous return.

### Species Differences in SAN Microstructure and Changes With Stretch

Species-specific responses of the SAN to stretch may also be modulated by differences in SAN structure and its stretch-induced alteration. At the epicardial surface of the non-stretched rabbit SAN, we observed aligned (Figure 4B), tightly crimped, and tortuous collagen fibers (Figure 4C), running parallel to the CT. In non-stretched mouse SAN, collagen fibers in the surface layer are also highly crimped and tortuous (Figure 4C) but poorly aligned (Figure 4B) and less dense, consistent with previous studies (Hao et al., 2011; Glukhov et al., 2015). During 40% stretch, collagen fibers in the surface layer of rabbit SAN became less tortuous (Figure 4C) although in mouse SAN fibers were rotated in the stretch direction (so became parallel to the CT; Figure 4B) without a change in crimp or tortuosity. In the middle layer of non-stretched SAN in both species, collagen fibers were crimped, tortuous (Figure 4C), and irregularly oriented (Figure 4B). This is in agreement with previous reports from rabbit (Bleeker et al., 1980). When SAN were stretched, collagen fibers aligned with the stretch direction (Figure 4B) and became less tortuous in both species. In the rabbit SAN, crimp of fibers in the middle layer was also reduced (Figure 4C). Interestingly, in the surface layers of the rabbit SAN and middle layers of the mouse SAN, collagen fiber tortuosity was reduced during stretch without a change in crimp wavelength. This apparent disparity suggests that, in addition to crimp, there is a “bowing” of collagen fibers, which is straightened first during stretch to reduce tortuosity before altering crimp wavelength. Overall, these results demonstrate that collagen fibers in rabbit and mouse SAN differ in their arrangement and in their response to stretch. In the rabbit SAN, collagen fibers predominantly straighten with stretch (with some rotation in the middle layer), and in the mouse SAN, collagen fibers predominantly rotate (with some straightening in the middle layer). Interestingly, despite their differing structures and their change with stretch, no species differences in SAN tissue stiffness or in average stress-strain relations were observed (Figure 2B).

Noting the potential importance of differences in collagen architecture of rabbit and mouse SAN, it is worth considering the cells responsible for its production. Like pacemaker cells in the SAN (Cooper et al., 2000), fibroblasts are mechano-sensitive and display *I*_*SAC,NS*_ (Stockbridge and French, 1988). Non-myocytes have recently been shown to electrically connect to myocytes in the ventricles (Quinn et al., 2016; Rubart et al., 2018) and to atrio-ventricular node cells (Hulsmans et al., 2017). This may be true also for pacemaker cells in the SAN (Camelliti et al., 2004), and it could account for at least some of the chronotropic response to stretch (Kohl et al., 1994). Presence, mechano-sensitivity, and electrical connectivity of cardiac non-myocytes may differ between species and contribute to the species dependence of BR responses to stretch. To comprehensively consider the role that collagen architecture might play in stretch responses, these findings should be followed up by experiments using biaxial or concentric stretch of isolated SAN or 3-D atrial volume manipulation (rather than in-plane or uniaxial stretch), where collagen fiber rotation will be less pronounced. This would also be closer to the stretch experienced by the SAN *in vivo*.

### Species Differences in SAN Nanostructure and Changes With Stretch

At the subcellular level, we observed an abundance of caveolae in non-stretched SAN cells (Figures 5A,B) as reported previously for rabbit (Bleeker et al., 1980). Caveolar density in the absence of stretch was twice as high in rabbit compared to mouse SAN, and the membrane area contained in caveolae adds an additional 117% of surface sarcolemma in rabbit and 66% in mouse when compared to a smoothly traced cell surface outline (in other words, membrane contained within caveolae constitutes roughly 54 and 40% of total surface sarcolemma in mice and rabbit, respectively). Cell surface membranes were less undulated in non-stretched SAN of rabbit compared to mouse. Upon stretch, surface membranes were flattened (Figure 5A: compare green and blue surfaces in the representative models of juxtaposed cells at baseline and during stretch; Figure 5D for quantitative data) and caveolar density was reduced in both species to the extent that differences were no longer apparent (Figure 5C). Membrane straightening and a reduction in caveolae, presumably by incorporation into the surface membrane (Kohl et al., 2003; Pfeiffer et al., 2014), is thought to relieve membrane tension as originally proposed for skeletal muscle (Dulhunty and Franzini-Armstrong, 1975). In ventricular myocytes, caveolar membrane integration into the surface sarcolemma has been shown to also alter passive electrophysiological membrane properties (increased capacity), reducing conduction velocity in response to stretch (Pfeiffer et al., 2014). Whether this mechanism contributes to stretch-induced changes in BR is unclear although it may be involved in stretch-mediated ion channel activation (Egorov et al., 2019). As chemical disruption of caveolar integrity in SAN cells alters pacemaking (Lang and Glukhov, 2018), it is not unreasonable to suspect that reconfiguration of caveolae by stretch, altering spatial compartmentalization of important signaling pathways, may affect SAN pacemaking. Whether or not this occurs on a beat-by-beat basis remains to be determined as the temporal offset between stretch and onset of tissue fixation – in our experiments about 1–2 s – is at least an order of magnitude slower than normal BR and any associated mechanical changes.

Despite more undulated surface membranes of non-stretched mouse SAN, given the lower presence of caveolae, murine cells have less spare membrane reserves to relieve membrane tension during stretch, so stress (rather than strain) could be a primary input parameter for mechano-sensitive responses in that species. This differs from rabbit and may be a result of adaptation to different intrinsic BR in the two species. At the high BR of mouse hearts, stroke volume will be relatively small compared to rabbit hearts with a much lower BR. Given that the relation between the changes in surface area and volume of a cardiac chamber, simplistically approximating it as a sphere, is linear (as surface area = 3 × volume/radius), small volume changes will result in small changes of surface area in the mouse and, therefore, less SAN stretch than in rabbit. This could involve a reduced requirement for “spare membrane” in mouse compared to rabbit.

### Species Differences in Stretch Responsiveness and SAN Stiffness

Even though the passive stress-strain relationships of rabbit and mouse SAN tissue were not significantly different, the difference in the relation of the change in BR to tissue stiffness suggests that pacemaker cells in the SAN of rabbit and mouse may experience or respond to different key mechanical stimuli during tissue stretch. In rabbit, stretch-induced changes in SAN BR appear to correlate with the amount of strain. This would be in keeping with our observation that stretch of rabbit SAN straightens collagen fibers at the epicardial surface and intramurally, which would result in stretch of interspersed pacemaker cells (Rego et al., 2016). In contrast, collagen fibers in mouse SAN rotate in the stretch direction but maintain their crimp wavelength, thereby potentially protecting associated pacemaker cells from themselves being stretched. Again, this should be assessed in future studies with biaxial, concentric, or *in situ* mechanical stimulation.

### The Importance of SAN Background Electrophysiology for the Chronotropic Response to Stretch

The difference in the chronotropic response to SAN stretch in rabbit (increase in BR) versus mouse (varied responses, including decreased BR) may be explained conceptionally by differences in their SAN AP shape with respect to *E*_*SAC,NS*_ (as described in the Introduction and schematically illustrated in Figure 3A). If applicable, an increased plateau duration of mouse SAN AP at unchanged baseline BR should shift the stretch-induced change in BR toward positive chronotropy. This effect was indeed observed in our study, using 4-AP at concentrations that had previously been shown to lengthen the plateau of mouse SAN AP by up to 50% without affecting the slope of final SAN repolarization or spontaneous diastolic depolarization (Golovko et al., 2015). This was observed in all cells tested, and the response disappeared upon washout, supporting the critical importance of intrinsic AP morphology for the chronotropic response of the SAN to stretch. Computational simulations, using an “out-of-the-box” model^1^, failed to reproduce the 4-AP induced shift toward an actual positive chronotropic response to stretch. It did reproduce, however, main features, such as the 4-AP induced prolongation of APD_20_ in the absence of changes in BR. This changed the chronotropic response of the SAN cell model to stretch from a reduction in BR (by 10%) to a much less pronounced effect (reduction by 3%; Supplementary Figure S5). This “positive shift” in the BR response to stretch after block of rapidly activating potassium currents agrees with the notion that AP shape matters for the extent and direction of the SAN stretch response.

That said, activation of *I*_*SAC,NS*_ by cyclic stretch exclusively during diastolic depolarization (to mimic the *in vivo* setting), would be expected to cause an even more pronounced and potentially species-independent increase in BR. So the Bainbridge response that speeds up initiation of the next wave of cardiac excitation if venous return from the systemic circulation is high may well be active in all species – another relevant target for follow-up experimental research, using dynamic stretch protocols.

### Study Limitations

The general anatomy of rabbit and mouse SAN is principally similar, and we made every effort to prepare the SAN from these two species and from each individual heart within each species in a matching manner. The fact that the “cut tissue” surface on the auricular side of the SAN would have been further away from the SAN in mouse compared to rabbit (2–3 mm instead of 1–2 mm) may have contributed to interspecies (but not to intersubject) differences in the chronotropic response to stretch. That said, the steady (no significant change over 2 h), low-variability, and relatively high (for isolated tissue) spontaneous BR of preparations indicate relatively well-sustained background activity of the SAN. Furthermore, although our 4-AP results support the idea that AP morphology is indeed a critical factor for differences in the directionality of chronotropic responses, this hypothesis has not been corroborated directly in the current study. This would have required AP assessment by microelectrode-based or optical voltage mapping from the *leading* SAN pacemaker site (which may shift during stretch) yet still be unable to address an ill-posed problem (in the presence of electrotonic entrainment of individual SAN cells). For this reason, we targeted ensemble properties using a pharmacological intervention known to modify SAN AP shape, but not duration. Although our computational simulations of 4-AP application support the quantitative plausibility of the proposed explanation, they do not prove it (Quinn and Kohl, 2011). It is difficult to ascertain, based on the type of data available and the constraints of the model, whether underlying mechanisms indeed match one another (for example, we are unable to relate the amount of SAC_*NS*_ activation in the experiments and model, and we cannot confirm the SAN AP shape in the leading pacemaker site of the preparation, which furthermore may change during stretch). This is compounded by the fact that the computational mouse SAN cell AP model used depicts only one of many AP morphologies occurring in the mouse SAN. Finally, it is important to note that the current study should not be used to construe a lack of physiological relevance of stretch for murine SAN BR regulation as this will require further work using cyclic stretch, applied during right atrial diastole.

## Conclusion

The current study illustrates the potential importance of SAN structural, mechanical, and background electrophysiological properties for the magnitude and direction of the chronotropic response to stretch in rabbit and mouse. A thorough understanding of the effects of the biophysical environment on SAN pacemaking is important for basic science, conceptual and mechanistic modeling with potentially important implications for further research into SAN dysfunction in the aging and/or fibrotic heart.

## Data Availability Statement

The datasets generated for this study are available on request to the corresponding authors.

## Ethics Statement

The animal study was reviewed and approved by the Dalhousie University Committee for Laboratory Animals or the local Institutional Animal Care and Use Committee in Freiburg, Germany (Regierungspraesidium Freiburg, X-16/10R).

## Author Contributions

EM contributed to the design of the study, performed SN stretch experiments, analyzed data, and drafted the manuscript. JM performed light imaging work and analyzed data. JG developed image analysis tools for collagen quantification and analyzed data. AR performed the computational simulations. SW contributed to the design of the study, developed image analysis tools for collagen quantification, and revised the manuscript. AT contributed to the design of the study, performed sample preparation, and revised the manuscript. PK contributed to the design of the study, performed sample preparation, and revised the manuscript. ER-Z contributed to the design of the study, performed light and electron imaging work, analyzed data, and revised the manuscript. TAQ contributed to the design of the study and revised the manuscript. All authors approved the final submission.

## Conflict of Interest

The authors declare that the research was conducted in the absence of any commercial or financial relationships that could be construed as a potential conflict of interest.

## Footnotes

1 We specifically abstained from “tweaking” the model even though the simulated SAN AP shape differs from that observed, for example, by Golovko et al. (2015), as to support replication and to avoid unintended user bias. The principally matching direction of 4-AP induced changes is, under these conditions, a conservative estimate of responses.

## Supplementary Material

FIGURE S1 Representative measurements of force and beating rate (BR) during sinoatrial node (SAN) stretch from the rabbit (**left**) and mouse, showing both a stretch that caused a decrease (**middle**) and a stretch that caused an increase (**right**) in BR.

FIGURE S2 **(A)** Change in BR of individual rabbit (**left**) and mouse (**middle**) SAN upon strain application of increasing magnitude and of individual mouse SAN (**right**) during application of various concentrations of 4-aminopyridine (4-AP) and after 20 min of washout. Each color represents an individual preparation (which are different in each panel).

FIGURE S3 **(A)** The relationship between change in BR of rabbit (**left**) and mouse (**right**) SAN with application of stretch of increasing magnitude and the baseline BR before stretch. **(B)** Comparison (green boxes cover matching areas in BR space) of the above relationship with the experiments by Cooper and Kohl (2005) in guinea pig and mouse SAN (at 27 or 37°C).

FIGURE S4 The relationship between the change in BR of mouse SAN upon application of 40% stretch and the baseline BR before stretch during exposure to various concentrations of 4-AP and after 20 min of washout.

FIGURE S5 Computational simulations of mouse SAN cell stretch with (“4-AP”) and without (“Control”) reduction of rapidly activating potassium currents (complete block of transient outward potassium current and 25% reduction in delayed rectifier potassium current) using the mouse SAN cell action potential model of Kharche et al. (2011). *E*_*SAC,NS*_, reversal potential of *I*_*SAC,NS*_.

## Abbreviations

4-AP: 4-aminopyridine
AP: action potential
BR: beating rate
CT: *Crista terminalis*
*E*_*SAC,NS*_: reversal potential of the cation non-selective stretch-activated channel
ET: electron tomography
*I*_*SAC,NS*_: cation non-selective stretch activated current
IVC: inferior (caudal) *vena cava*
SAN: sinoatrial node
SHGM: second-harmonic generation microscopy
SVC: superior (cranial) *vena cava*.
