# Supplementary figures and images for: Sinoatrial Node Structure, Mechanics, Electrophysiology and the Chronotropic Response to Stretch in Rabbit and Mouse
Source: Front Physiol. 2020 Jul 22;11:809. doi: 10.3389/fphys.2020.00809 (PMC7388775; doi:10.3389/fphys.2020.00809)

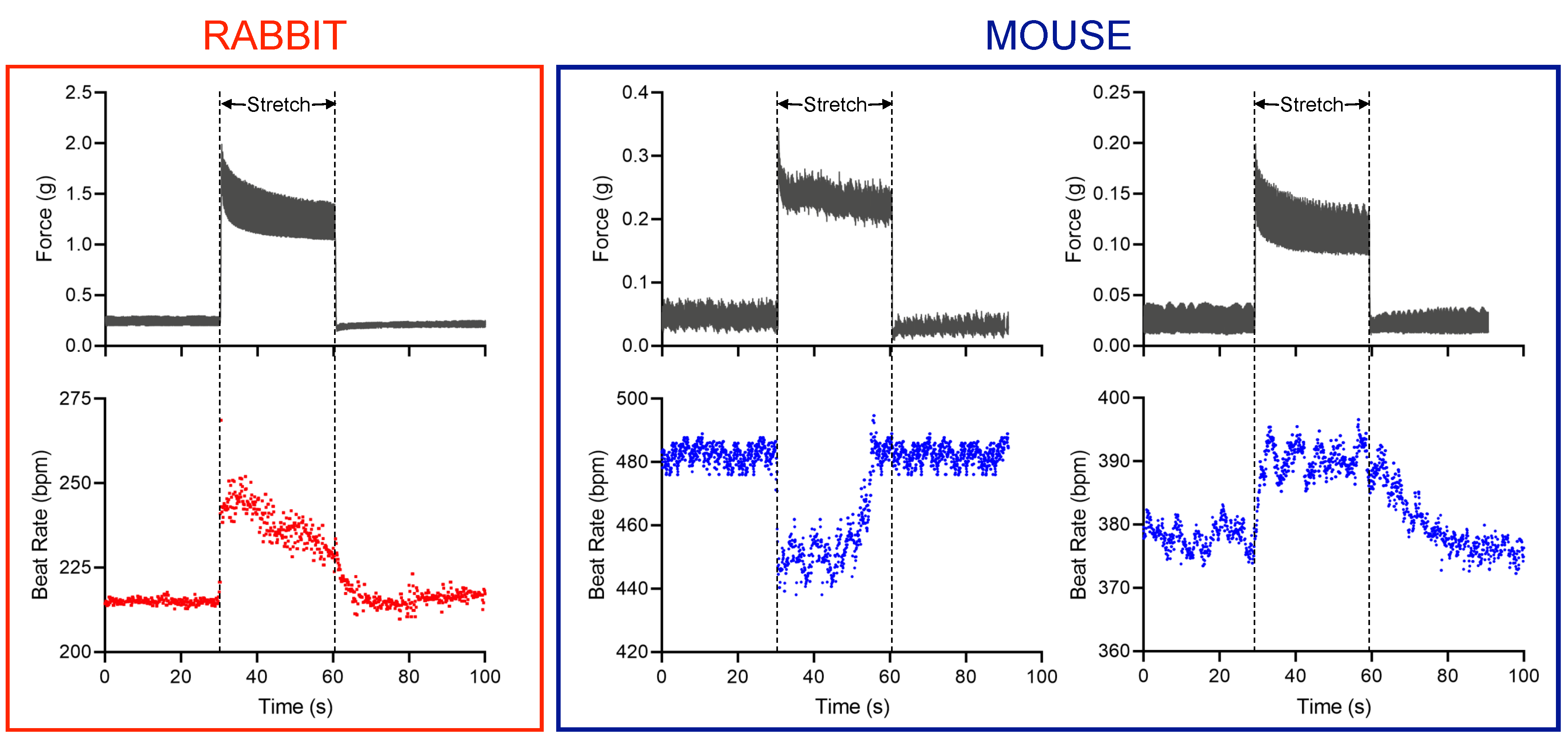

Supplement: FIGURE S1 — Representative measurements of force and beating rate (BR) during sinoatrial node (SAN) stretch from the rabbit (left) and mouse, showing both a stretch that caused a decrease (middle) and a stretch that caused an increase (right) in BR. [file Image_1.TIF]

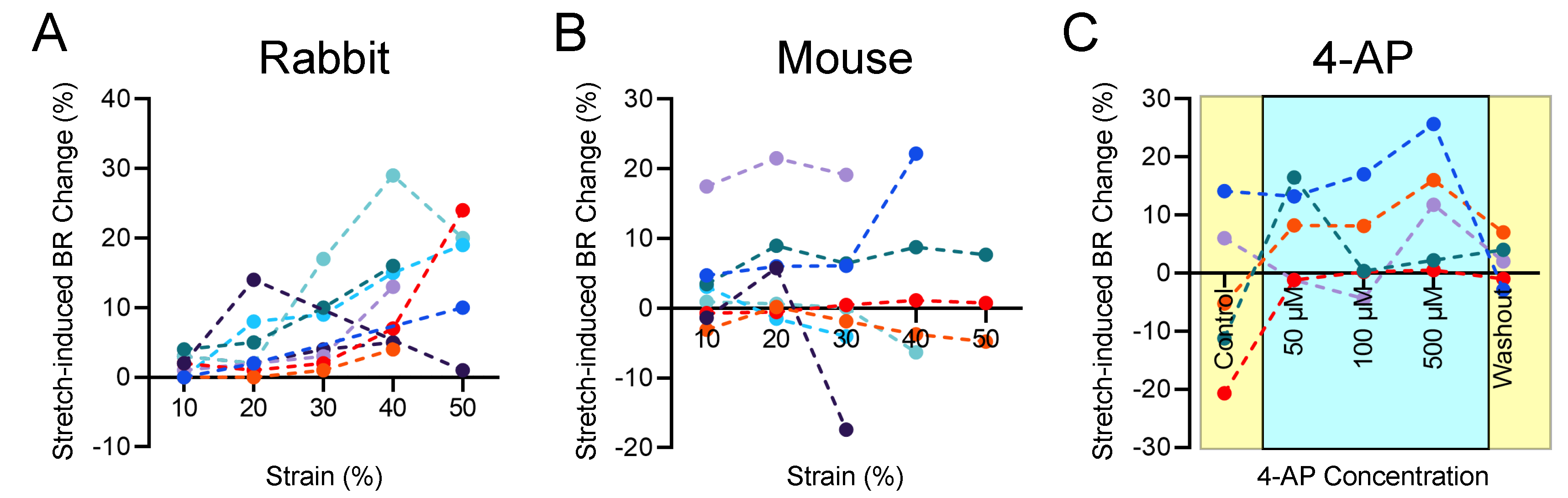

Supplement: FIGURE S2 — (A) Change in BR of individual rabbit (left) and mouse (middle) SAN upon strain application of increasing magnitude and of individual mouse SAN (right) during application of various concentrations of 4-aminopyridine (4-AP) and after 20 min of washout. Each color represents an individual preparation (which are different in each panel). [file Image_2.TIF]

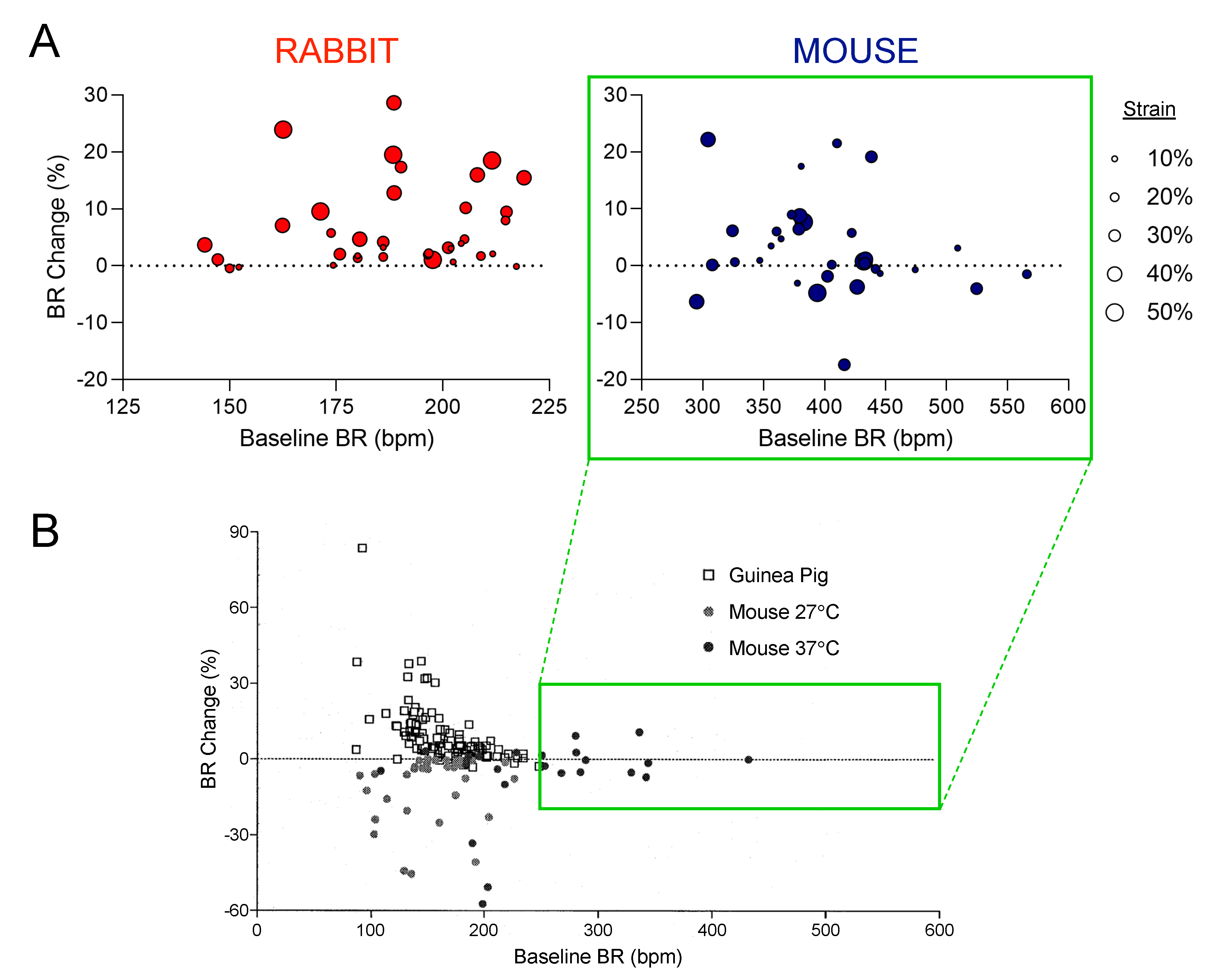

Supplement: FIGURE S3 — (A) The relationship between change in BR of rabbit (left) and mouse (right) SAN with application of stretch of increasing magnitude and the baseline BR before stretch. (B) Comparison (green boxes cover matching areas in BR space) of the above relationship with the experiments by Cooper and Kohl (2005) in guinea pig and mouse SAN (at 27 or 37°C). [file Image_3.TIF]

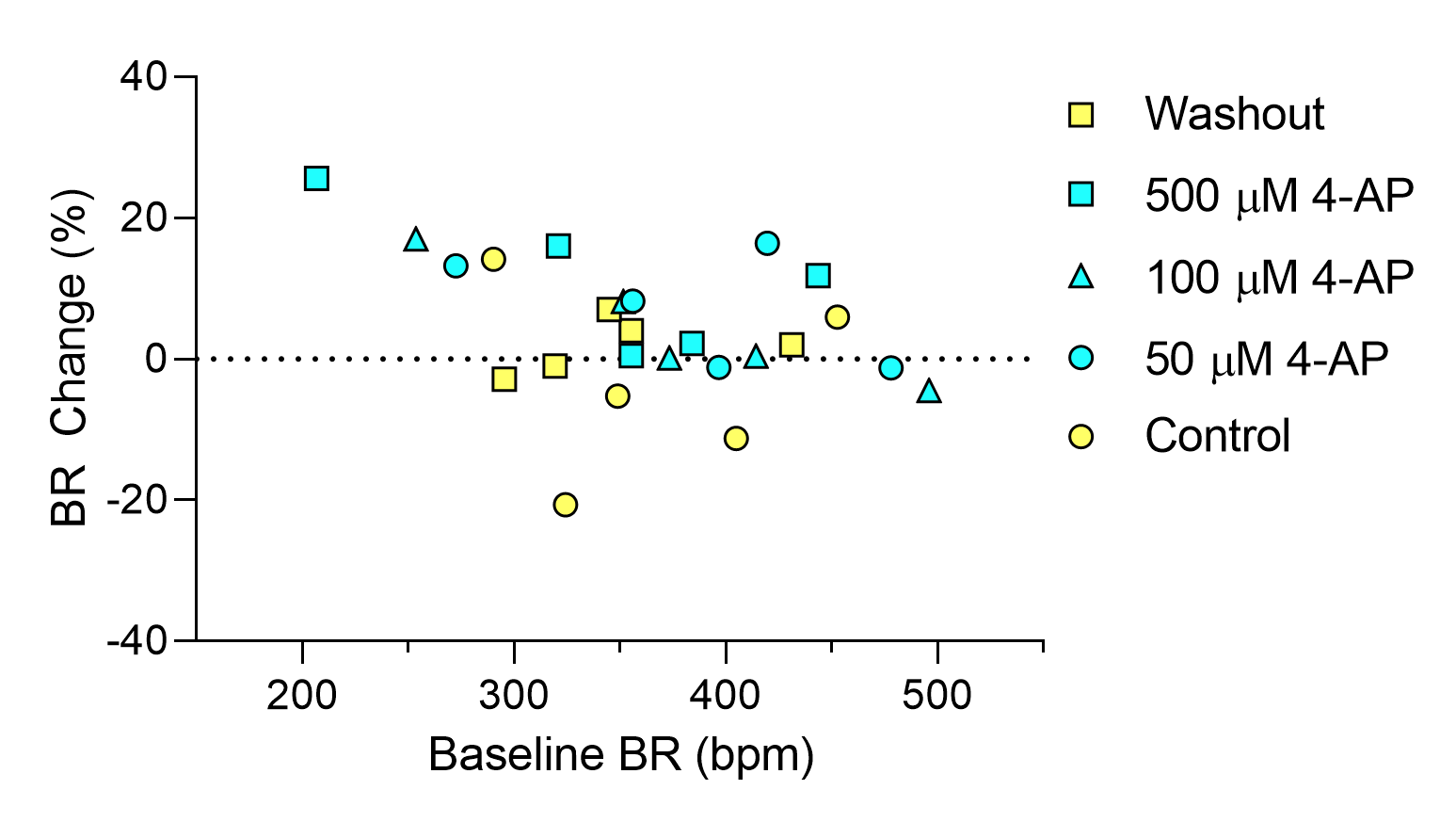

Supplement: FIGURE S4 — The relationship between the change in BR of mouse SAN upon application of 40% stretch and the baseline BR before stretch during exposure to various concentrations of 4-AP and after 20 min of washout. [file Image_4.TIF]

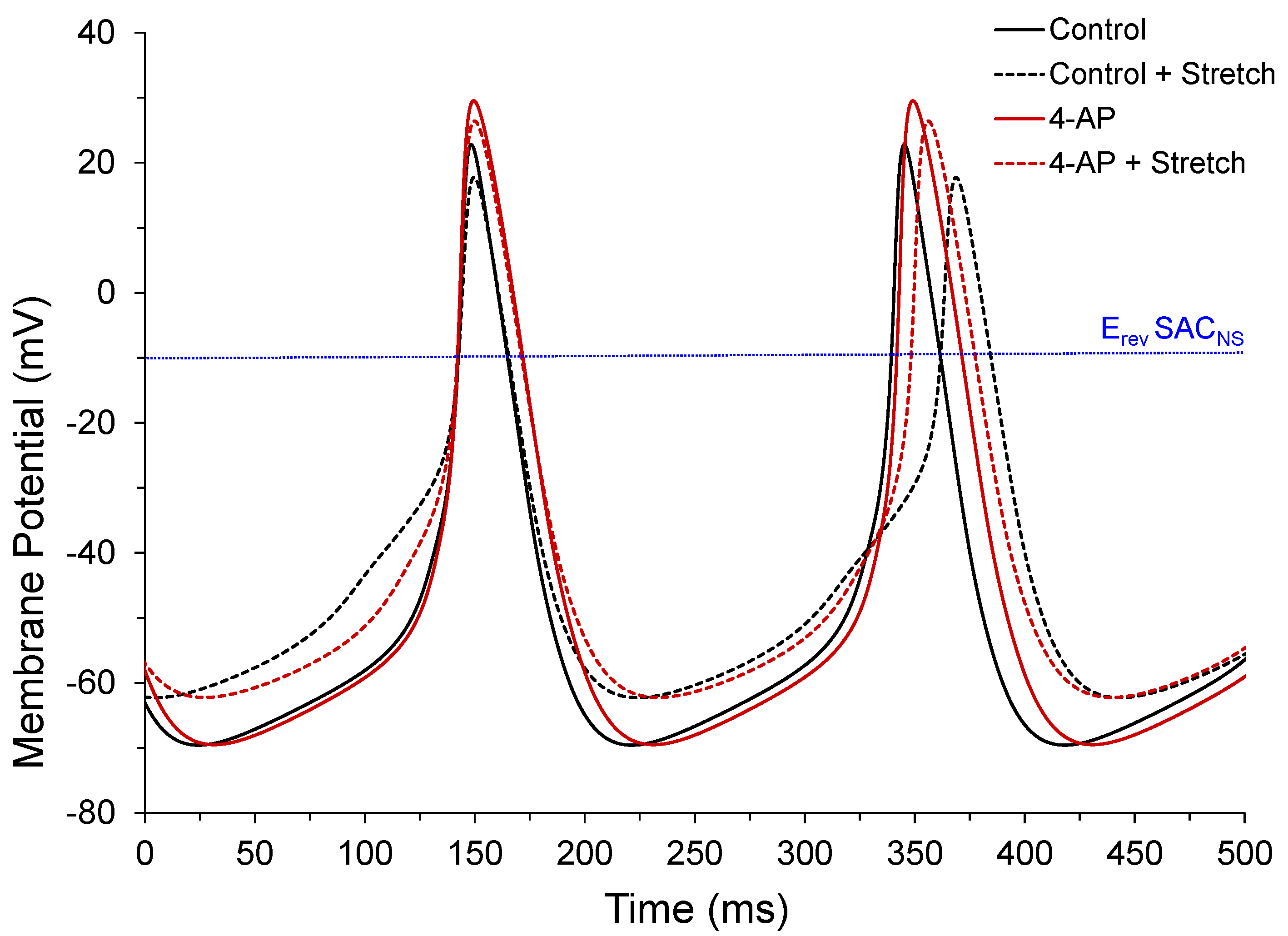

Supplement: FIGURE S5 — Computational simulations of mouse SAN cell stretch with (“4-AP”) and without (“Control”) reduction of rapidly activating potassium currents (complete block of transient outward potassium current and 25% reduction in delayed rectifier potassium current) using the mouse SAN cell action potential model of Kharche et al. (2011). ESAC,NS, reversal potential of ISAC,NS. [file Image_5.TIF]
